# Supplementary material for: A Critical View of the Application of the APEX Software (Aqueous Photochemistry of Environmentally-Occurring Xenobiotics) to Predict Photoreaction Kinetics in Surface Freshwaters
Source: Molecules. 2019 Dec 18;25(1):9. doi: 10.3390/molecules25010009 (PMC7017383; doi:10.3390/molecules25010009)
Supplement: Supplementary file 1 [file molecules-25-00009-s001.zip › 3.2.4_gcc-4.4.0.zip!/3.2.4_gcc-4.4.0/3.2.4_gcc-4.4.0/doc/octave/html/interpreter/Data-sources-in-object-groups.html]

Data sources in object groups - Untitled


Next: Area series,
Up: Object Groups


---

##### 15.2.8.1 Data sources in object groups

All of the group objects contain data source parameters. There are
string parameters that contain an expression that is evaluated to update
the relevant data property of the group when the `refreshdata`
function is called.

— Function File: **refreshdata** ()  
— Function File: **refreshdata** (h)  
— Function File: **refreshdata** (h, workspace)  
> Evaluate any datasource properties of the current figure and update
> the plot if the corresponding data has changed. If called with one or more
> arguments h is a scalar or array of figure handles to refresh. The
> optional second argument workspace can take the following values.
>
> `"base"`: Evaluate the datasource properties in the base workspace. (default). `"caller"`: Evaluate the datasource properties in the workspace of the function that called `refreshdata`.
>
> An example of the use of `refreshdata` is:
>
> ```
>           x = 0:0.1:10;
>           y = sin (x);
>           plot (x, y, "ydatasource", "y");
>           for i = 1 : 100
>             pause(0.1)
>             y = sin (x + 0.1 * i);
>             refreshdata();
>           endfor
> ```
